# Preliminary study of tumor heterogeneity in imaging predicts two year survival in pancreatic cancer patients

**DOI:** 10.1371/journal.pone.0188022

**Published:** 2017-12-07

**Authors:** Jayasree Chakraborty, Liana Langdon-Embry, Kristen M. Cunanan, Joanna G. Escalon, Peter J. Allen, Maeve A. Lowery, Eileen M. O’Reilly, Mithat Gönen, Richard G. Do, Amber L. Simpson

**Affiliations:** 1 Department of Surgery, Memorial Sloan Kettering Cancer Center, New York, New York, United States of America; 2 Department of Epidemiology and Biostatistics, Memorial Sloan Kettering Cancer Center, New York, United States of America; 3 Department of Radiology, Memorial Sloan Kettering Cancer Center, New York, United States of America; 4 Department of Medicine, Memorial Sloan Kettering Cancer Center, New York, United States of America; University of Nebraska Medical Center, UNITED STATES

## Abstract

Pancreatic ductal adenocarcinoma (PDAC) is one of the most lethal cancers in the United States with a five-year survival rate of 7.2% for all stages. Although surgical resection is the only curative treatment, currently we are unable to differentiate between resectable patients with occult metastatic disease from those with potentially curable disease. Identification of patients with poor prognosis via early classification would help in initial management including the use of neoadjuvant chemotherapy or radiation, or in the choice of postoperative adjuvant therapy. PDAC ranges in appearance from homogeneously isoattenuating masses to heterogeneously hypovascular tumors on CT images; hence, we hypothesize that heterogeneity reflects underlying differences at the histologic or genetic level and will therefore correlate with patient outcome. We quantify heterogeneity of PDAC with texture analysis to predict 2-year survival. Using fuzzy minimum-redundancy maximum-relevance feature selection and a naive Bayes classifier, the proposed features achieve an area under receiver operating characteristic curve (AUC) of 0.90 and accuracy (*Ac*) of 82.86% with the leave-one-image-out technique and an AUC of 0.80 and *Ac* of 75.0% with three-fold cross-validation. We conclude that texture analysis can be used to quantify heterogeneity in CT images to accurately predict 2-year survival in patients with pancreatic cancer. From these data, we infer differences in the biological evolution of pancreatic cancer subtypes measurable in imaging and identify opportunities for optimized patient selection for therapy.

## Introduction

Pancreatic ductal adenocarcinoma (PDAC) is the fourth leading cause of cancer-related death in the United States with more than 53,000 new diagnoses and 41,000 deaths expected in 2016 [1]. The 5-year survival rate for PDAC is low (7.2% for all stages) and the mortality rate is increasing. Surgery is the only curative treatment; however, only 10 − 20% of patients present with resectable disease and only 5 − 15% of these patients remain disease-free at 5 years [2]. Despite improvements in the understanding of pancreatic cancer, the impact of molecularly targeted therapies on outcome for PDAC patients has been limited [3]. Thus, novel prognostic markers are needed to improve management of PDAC.

The application and benefit of adjuvant and neoadjuvant chemotherapies are not well elucidated in this patient population [4–8] and also complicated by the fact that we are unable to distinguish between resectable PDAC patients with occult metastatic disease from those with potentially curable disease [9]. Identifying patients most likely to benefit from neoadjuvant therapy prior to treatment would improve selection of patients for curative surgery, e.g., those who should delay surgical resection for aggressive systemic treatment. Therefore, early classification of tumor aggressiveness may lead to changes in initial management including the use of neoadjuvant chemotherapy or radiation, or in the choice of postoperative adjuvant treatments [10]. Several surgical, pathological, clinical, and molecular factors have been investigated for prognostic significance [11–13] with radiographic and pathological factors showing potential in prognosis stratification. However, pathological variables are only available after resection and are therefore of little clinical benefit, and radiographic criteria (i.e., tumor volume) alone are not prognostic.

Recently, a consensus statement by the Society of Abdominal Radiology and the American Pancreatic Association highlighted the imaging heterogeneity of PDAC, outlining imaging reporting guidelines for this tumor [14]. These guidelines include morphologic descriptions of tumor appearance on computed tomography (CT) as hypoattenuating (areas of darker attenuation) or isoattenuating (areas of brighter attenuation), imaging properties that may predict the degree of PDAC differentiation [15] or the interaction of tumor cells and pancreatic stroma [16]. Limiting the description of PDAC to hypoattenuating or isoattenuating does not fully capture the range of heterogeneity in these tumors. Texture analysis is a well-established image processing technique that is an emerging methodology in oncologic imaging for quantifying tumor heterogeneity [17]. Studies have shown the potential of texture analysis for prognostication in a number of malignancies, including lung, breast, and prostate cancer [18]. Fundamentally, analysis of heterogeneity is based on perfusion changes, which reflect variations in the tumor microenvironment. In addition to enhancement patterns, texture analysis can reveal differences in cellular density in tumors matched to histologic findings [19] or distinguish benign and malignant tissues [20]. In pancreatic cancer, texture analysis was recently used to predict malignancy in pancreatic cysts (pre-cursor lesions to pancreatic cancer) [21] but to our knowledge, ours is the first work correlating texture analysis with survival in pancreas cancer. Others have related changes in Hounsfield units from the precontrast, arterial, and portal-venous phases in pancreas protocol scans can predict outcome [22].

We hypothesize that the imaging phenotypes of PDAC on CT reflect underlying differences at the histologic or genetic level, which correlate with patient outcome. We investigate texture to quantify differences among PDAC on pre-treatment portal venous phase CT to predict 2-year overall survival. The rationale for selecting 2-year survival is that patients surviving less than 2 years likely have occult metastatic disease and should undergo aggressive systemic therapy prior to surgery. Preliminary analysis of these data for predicting survival at 5 years was presented at SPIE Medical Imaging [23]. This paper extends the imaging feature set to include new features and incorporates clinical variables into the prediction model.

## Materials and methods

**Fig 1:**
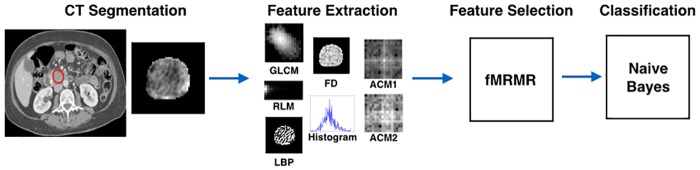
Schematic of the methods.

We investigate tumor texture as a prognostic factor prior to treatment based on the observation that PDAC has variable appearance, which may be associated with survival. We chose 2-year survival as the endpoint based on our own published data on the median survival of patients with pancreatic cancer who undergo resection [24]. Detailed description of the proposed methods is provided in the following section. A schematic of our methods is shown in Fig 1. Briefly, texture is quantified using many intensity and directional edge-based features extracted from the tumor region. Fuzzy minimum-redundancy maximum-relevance (fMRMR) feature selection identified features for incorporation into the naive Bayes classifier. Classification performance was evaluated using leave-one-image-out cross-validation. All methods were implemented using MATLAB version R2015a (Natick, MA, USA).

### Study design and patients

Patients signed an informed consent that covered review of medical records and studies for correlated research. The study was approved by the Institutional Review Board (IRB) of Memorial Sloan Kettering Cancer Center (MSKCC). Patients enrolled in a phase II clinical trial at our institution on the role of neoadjuvant chemotherapy in resectable PDAC (NCT00536874) were included in our retrospective analysis [25]; all patients signed IRB-approved consent forms for participation in this trial. As part of the clinical trial, all patients were untreated at the time of baseline CT imaging, the most common profile for patients newly diagnosed with pancreas cancer and thus generalizable to all PDAC patients. This is an ideal study population for texture analysis because patients were imaged with the same CT imaging protocol and prior to chemotherapy treatment, thus we control for factors that potentially influence texture features.

A waiver of Health Insurance Portability and Accountability Act authorization and informed consent was granted through Institutional Review Board approval to retrospectively analyze these data. Of the original thirty-eight patients, three were excluded from our study due to insufficient imaging. The remaining thirty-five patients were included in our analysis. Detailed description of patient selection and treatment characteristics of this cohort is reported by O’Reilly *et al*. [25]. Briefly, the selected patients were enrolled in the clinical trial between July 2007 and December 2011. The trial included resectable patients with age > 18 years and excluded all patients with borderline resectable or locally advanced pancreas adenocarcinoma. Neoadjuvant chemotherapy comprised four cycles of gemcitabine dosed at 1000 mg/m^2^ IV over 100 minutes and oxaliplatin 80 m/m^2^ IV over 2 hours, at every 2 weeks. After the completion of neoadjuvant therapy, eligible patients were resected within 2-6 weeks. All resected patients subsequently received 5 cycles of adjuvant gemcitabine (1000 mg/m^2^ IV over 30 minutes) for 15 doses.

### Imaging protocol

Patients underwent contrast-enhanced CT imaging as part of the clinical trial. The post-contrast CT images were acquired following the administration of 150 mL iodinated contrast (Omnipaque 300, GE Healthcare, New Jersey) at 4.0 mL/sec, on multidetector CT (Lightspeed 16 and VCT, GE Healthcare, Wisconsin). The scan parameters were as follows: pitch/table speed = 0.984-1.375/39.37-27.50 mm; autoMA 220-380; noise index 12.5-14; rotation time 0.7-0.8 ms; scan delay 80-85 s. The voxel size was [0.7324, 0.7324, 2.5] mm. For image analysis, axial slices reconstructed at each 2.5 mm interval were used.

### Image segmentation

**Fig 2:**
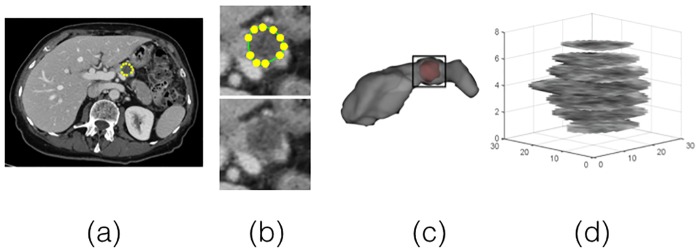
(a) Extracted CT slice after acquisition, (b) magnified view of tumor region with (top) and without (bottom) the manually drawn boundary, (c) 3-D view of manually segmented pancreas with tumor, (d) 2-D slices of tumor.

The tumor region was manually delineated by an experienced radiologist, blinded to clinical outcome, using Scout Liver (Pathfinder Technologies Inc., Nashville, TN) (Fig 2).

### Texture feature extraction

To quantify the tumor texture, 255 well-established first- and second-order intensity and edge-based features were extracted using gray-level co-occurrence matrices (GLCM) [26], run-length matrices (RLM) [27], local binary patterns (LBP) [28, 29], fractal dimension (FD) [30], intensity histogram (IH), and angle co-occurrence matrices (ACM) [31, 32]. A 2-D feature extraction technique was employed, where features are computed from each slice and averaged over the slices to provide a single value for the entire tumor.

GLCM encode the spatial distribution of pixels in a neighborhood of an image by computing the probability of occurrences of each pixel pair located at a specified distance and angle. To derive rotation invariant features, four matrices were calculated with angles 0°, 45°, 90°, and 135° with empirically selected distance *d* = 2 pixels and quantized intensity levels *N* = 16, averaged to form a single resultant matrix. Statistical features were extracted from the matrix as follows: 14 Haralick texture features, inertia, cluster shade, cluster prominence, Renyi entropy, and Tsallis entropy (*G*_1_ − *G*_19_) [26, 33, 34]. Renyi entropy and Tsallis entropy are defined as

$$G_{18}=\frac{1}{1-q}{\text{log}}_{2}\sum_{i=1}^{N}\sum_{j=1}^{N}{\left[GLCM\left(i,j\right)\right]}^{q},\;\;\;G_{19}=\frac{1}{1-r}\sum_{i=1}^{N}\sum_{j=1}^{N}{\left[GLCM\left(i,j\right)\right]}^{r},$$

where *q* and *r* are the order of Renyi and Tsallis entropies, empirically selected as 8 and 2, respectively.

Observing the persistent occurrence of long gray-level runs in coarser textures and short gray-level runs in finer textures, RLM were introduced, which quantify the coarseness of texture by counting number of consecutive pixels in a specific direction [27]. Similar to GLCM, RLM were calculated in four directions (0°, 45°, 90°, and 135°). Eleven features were derived from each matrix and averaged to obtain rotation invariant coarseness measures (*R*_1_ − *R*_11_) [27].

Based on the hypothesis that texture of an image has two components, pattern and strength, Ojala et al. [28, 29] introduced LBP to characterize the local textural patterns, which assign a value for each pixel by thresholding its 3 × 3 neighbors with the center pixel and computing the decimal value corresponding to the generated eight-bit stream. For the LBP-based features, we used the histograms of uniform LBP (ULBP) and rotation invariant ULBP (RI-ULBP) [29, 35, 36], two modified operators, which omit less often occurring non-uniform patterns and provide rotation invariant patterns, respectively. The statistical properties of these histograms as well as histograms of LBP, RI-LBP, and rotated LBP [37], such as standard deviation, skewness, kurtosis, and entropy, were also considered as features. Moreover, the rotation invariant LBP histogram Fourier features were extracted by applying discrete Fourier transform on LBP-histogram [38]. A set of 128 LBP features are thus constructed, which contains 59 ULBP (*L*_1_ − *L*_59_), 10 RI-ULBP (*L*_60_ − *L*_69_), 21 statistical (*L*_70_ − *L*_90_), and 38 frequency (*L*_91_ − *L*_128_) descriptors.

Several techniques have been proposed to derive the FD of an image [39, 40], which measures image self-similarity. In the present study, the segmentation-based fractal texture analysis (SFTA) was employed to explore the segmented textural patterns [41]. SFTA decomposes the image into a set of 16 binary images and computes the FD (FD1) from borders of each of the segmented regions using a box-counting method, which generates 48 features (*F*1_1_ − *F*1_48_). The popular differential box-counting (DBC) algorithm [39], with 7 × 7 neighbors, was also applied over each pixel of the image to obtain an FD image (FD2). The DBC method was chosen due to its superior performance over the Brownian motion algorithm [39]. The maximum and average value of mean, standard deviation, and lacunarity extracted from FD images over all the slices were considered as another set of features (*F*2_1_ − *F*2_6_).

Five elemental first-order statistical features, mean, standard deviation, skewness, kurtosis, and entropy, were computed using the intensity-histogram (*I*_1_ − *I*_5_).

To characterize the directional edge patterns of the tumor, two ACMs [31, 32, 42] were computed based on joint occurrences of the texture orientation angles using gradient information of the tumor, extracted with a Sobel operator of size 3 × 3. While applying the Sobel operator to compute the gradient information within the tumor region, we ignored the processing of boundary pixels to avoid any ambiguity caused by pixels outside the tumor region. The (*i*, *j*)^th^ element of ACM_(*l*,*θ*)_ represents the probability of occurrence of the pair of angles (*i*, *j*) with separation of distance *l* and angle *θ*. The first ACM (*ACM*1) is computed using gradient orientation, whereas the second ACM (*ACM*2) is formed using gradient orientation as well as magnitude. These can be written as

$$\text{ACM}1_{\left(l,\theta \right)}\left(i,j\right)=\frac{S_{a}\left(i,j\right)}{\sum_{i=1}^{N_{\theta }}\sum_{j=1}^{N_{\theta }}S_{a}\left(i,j\right)},\;\;\text{ACM}2_{\left(l,\theta \right)}\left(i,j\right)=\frac{S_{m}\left(i,j\right)}{\sum_{i=1}^{N_{\theta }}\sum_{j=1}^{N_{\theta }}S_{m}\left(i,j\right)},$$ (1)

where *S*_*a*_(*i*, *j*) and *S*_*m*_(*i*, *j*) are the number of occurrences and the sum of gradient magnitude responses, respectively, of all pixel-pairs with gradient angle *i* and *j*, separated by (*l*, *θ*); *N*_*θ*_ is the number of quantized angle levels. In this study, *l* and *N*_*θ*_ were empirically selected as 1 and 8, respectively. The same features as computed for GLCM were extracted from the ACMs. The features are rotation invariant after averaging over four directions 0°, 45°, 90°, and 135°.

**Fig 3:**
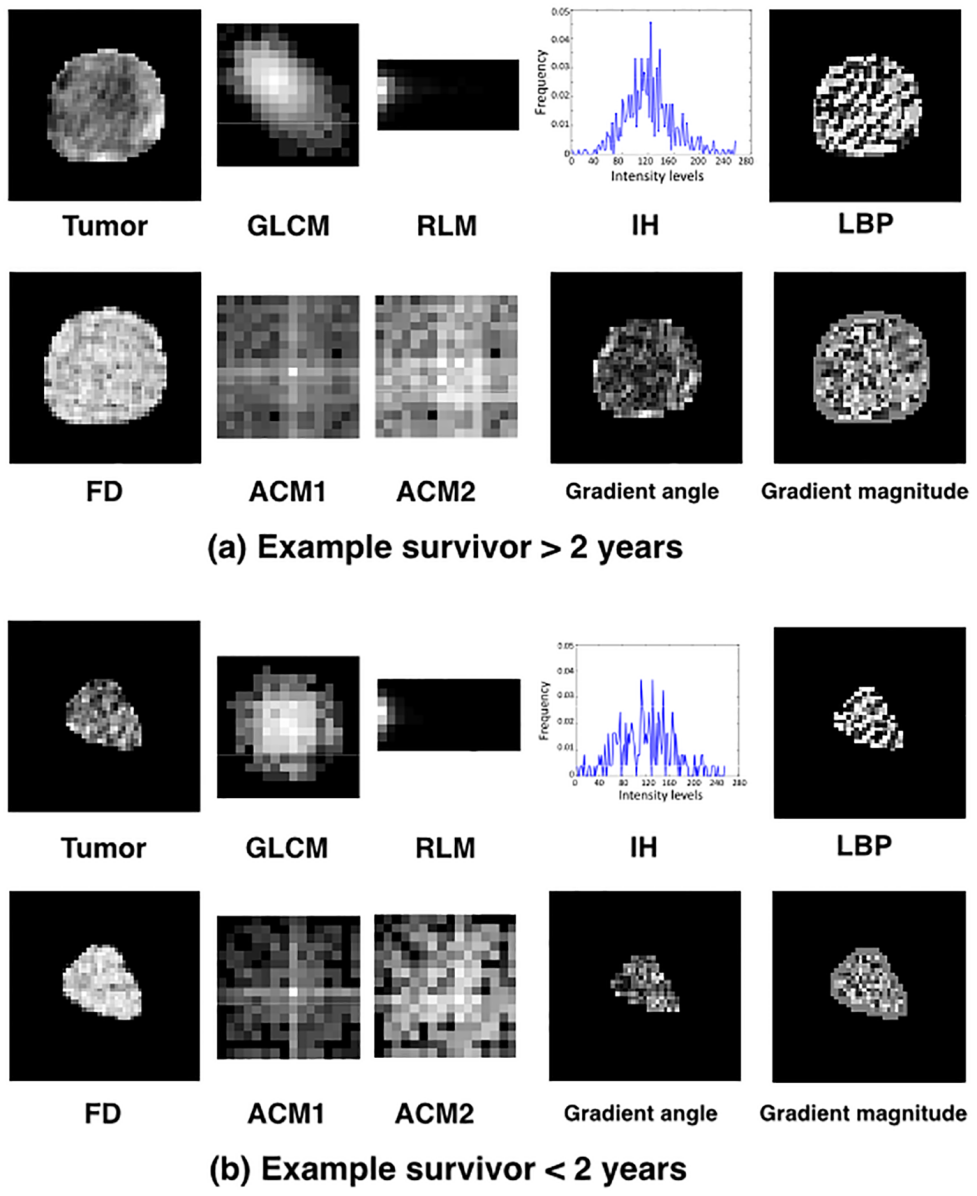
Exemplar tumors with rendered texture features displayed by converting data into gray levels with range [0, 255]. Resultant matrices rendered from GLCM, RLM, ACM1, and ACM2. Histogram used in the derivation of IH features. LBP and FD values at each pixel. Gradient angle computed with Sobel operator on each pixel used in ACM1 and ACM2 features. Gradient magnitude computed with Sobel operator on each pixel used in ACM2 features.

Derived features are listed in S1 Table. Examples of tumors with rendered texture representation for two patients with overall survival greater than and less than 2 years are shown in Fig 3.

### Feature selection

Feature selection was used to identify features with sufficient discriminatory power and to avoid overfitting of the prediction model with 255 imaging features and only 35 patients for model construction. We used fMRMR feature selection technique due to its simplicity and comparable performance with other methods including stepwise logistic regression, Fisher score, and wrapper [43–46]. Important features were selected from only the training set to avoid the effect of bias. The fMRMR technique is described in Algorithm 1. An incremental search technique is incorporated with fMRMR where features are selected incrementally, based on their relevancy as measured by fuzzy mutual information of a feature within the classes and redundancy as measured by averaging fuzzy mutual information of the feature with the already selected features using Eq 2. This iterative process creates a total of *D* sequential feature sets (*S*_1_, *S*_2_,…,*S*_*D*_) such that (*S*_1_ ⊂ *S*_2_ ⊂ … ⊂ *S*_*D*−1_ ⊂ *S*_*D*_), where *D* is the dimension of features in descending order of importance. We used forward selection to select the optimal number of features using classification error from the naive Bayes classifier consistent with related work [45].

**Algorithm 1** fMRMR feature selection

**Input:** Set of training data with D-dimensional feature vectors *F* = {*f*_1_, *f*_2_,…,*f*_*D*_}.

**Output:** Optimum features set $F^{*}=\left\{f_{1}^{r},f_{2}^{r},...,f_{O}^{r}\right\}\in F$

**begin**

1. For m = 1 to D

 i. With the incremental fMRMR algorithm select feature using the following condition:

$$J\left(f_{j}\right)=\underset{f_{j}\in F-F_{m-1}^{r}}{\text{max}}[MI\left(f_{j};c\right)-\frac{1}{m-1}\sum_{f_{i}\in F_{m-1}^{r}}MI\left(f_{j};f_{i}\right)],$$ (2)

  where *MI* represents fuzzy mutual information between two variables, *c* = [0, 1] represents the class vector, and $F_{m-1}^{r}$ is the set of already selected features.

 ii. This creates a set of features, $F_{m}^{r}=\left\{f_{1}^{r},f_{2}^{r},...,f_{m}^{r}\right\}\subset F$ and $⊃F_{m-1}^{r}$, ranked according to their importance.

2. Determine optimal size (*O*) of candidate feature set $\left(F_{O}^{r}\right)$ with leave-one-image-out technique that provides minimum classification error:

$$F^{*}=F_{O}^{r}=\left\{f_{1}^{r},f_{2}^{r},...,f_{O}^{r}\right\},\;\text{where}\;O=\text{a}rg\min_{k\in [1,\;D]}\;\left\{e_{k}\right\},$$

where *e*_*k*_ = classification error with feature set *S*_*k*_.

**end**

### Classification and evaluation

To evaluate the predictive value of the selected features for 2-year survival, two classifiers were implemented: a naive Bayes classifier [47] where the conditional probability of features for a given class is assumed to follow a Gaussian distribution and a support vector machine (SVM) classifier.

Due to the small size of the dataset, splitting of data strictly into training and testing sets was not feasible. To minimize data overfitting, cross-validation is an effective strategy of analyzing the performance [48]. We used leave-one-image-out and three-fold cross-validation, consistent with the literature [49]. The leave-one-image-out method is the extreme form of cross-validation, where one sample is used for testing and the remaining observations are used to train the model. This is repeated until all images are explored as test data. In three-fold cross-validation, observations are randomly divided into three groups. Each of the groups is used exactly once as the test set while the others are used for training. To reduce the performance variability, three-fold cross-validation was repeated twenty times, each with a different partition, and the results were averaged over all iterations.

Evaluation of the predictive performance of texture features for 2-year survival is described using receiver operating characteristic (ROC) curve, area under ROC curve (AUC), and classification accuracy (*Ac*) with corresponding sensitivity (*Sn*) and specificity (*Sp*) obtained by applying a threshold of 0.5 on classifier outcome, applying equal prior probabilities to both classes.

### Survival analysis

Analysis of clinical variables with respect to overall survival was performed using Statistical Software for the Social Sciences (SPSS version 22.0, IBM, Armonk, New York, USA). A p-value of 0.05 or less was considered significant. Univariable overall survival analysis was carried out with Kaplan-Meier statistics (log-rank test) for all binary clinical variables and Cox proportional hazards model for continuous variables. Univariable analysis of associations in continuous and binary clinical variables between patient surviving less than 2 years and patients surviving greater than 2 years were undertaken with Mann Whitney and Pearson’s chi-squared tests, respectively.

## Results

Thirty-five patients were included in the analysis. Patient demographics are summarized in Table 1. The median overall survival for this cohort was 29 months. ECOG performance status (p<0.01) and tumor location (p<0.05) were correlated with overall survival but gender, age, CA 19-9 level, and tumor size were not. No clinical variables were significantly different in patients surviving less than and greater than 2 years (Table 1), likely due to the small sample size.

**Table 1:** Correlation of pre-treatment patient factors with survival.

Characteristic	All (n = 35)	Survival < 2 years (n = 20)	Survival ≥ 2 years (n = 15)	p-value
Sex, n (%)				
Male	20 (57)	9 (26)	11 (31)	p = 0.158
Female	15 (43)	11 (31)	4 (11)	
Age, median (range), yr	69 (40-87)	67 (40-79)	71 (43-87)	p = 0.107
ECOG performance status, n (%)				
ECOG 0	13 (37)	10 (29)	3 (9)	p = 0.139
ECOG 1	22 (63)	10 (29)	12 (34)	
Primary pancreas tumor location, n (%)				
Head/neck	29 (83)	15 (43)	14 (40)	p = 0.184
Body	2 (6)	1 (3)	1 (3)	
Tail	4 (11)	4 (11)	0 (0)	
CA 19-9 level, median (range), U/mL	110 (3-3816)	89 (23-1687)	242 (3-3816)	p = 0.191
Tumor volume, median (range), mm^3^	6 (1-18)	4 (1-12)	7 (1-17)	p = 0.107

**Fig 4:**
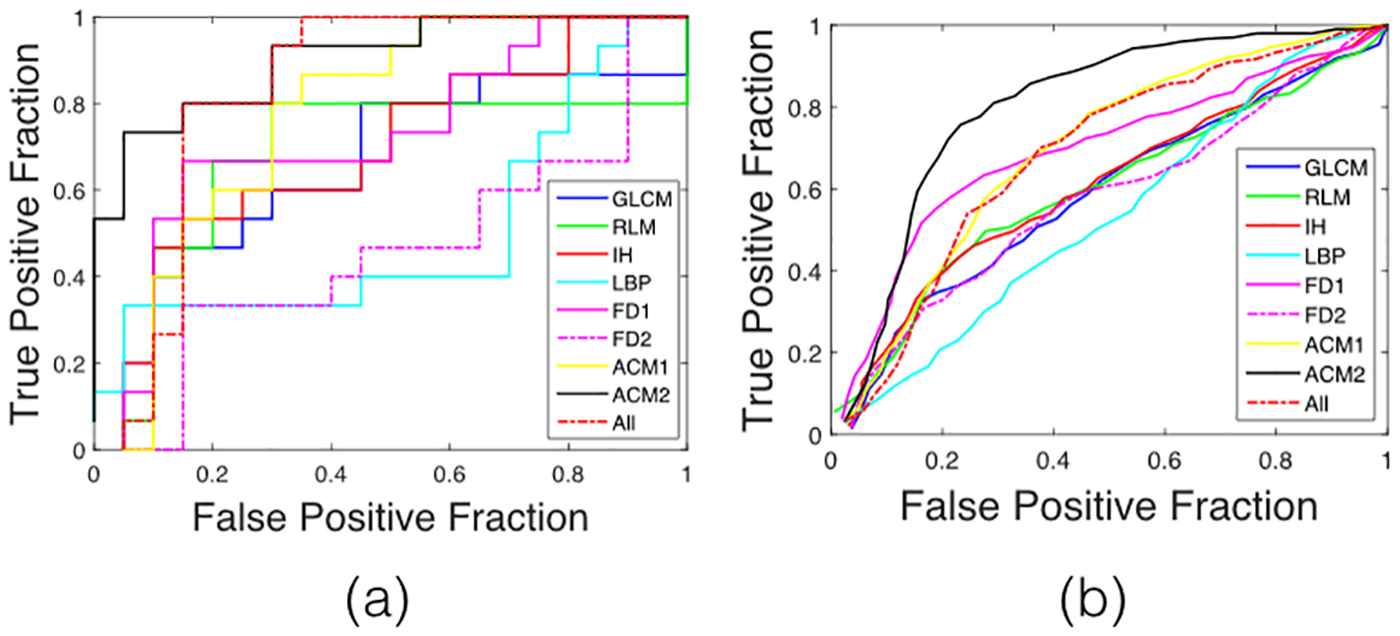
ROC curves obtained with different feature sets extracted from the tumor region using (a) leave-one-image-out and (b) three-fold cross-validation techniques.

Our analysis indicates that texture features predict 2-year survival in patients with PDAC. Further, combinations of texture features provide better discriminatory power. We investigated the performance of each type of feature (GLCM, RLM, IH, LBP, FD, ACM1, ACM2) as well as the combination of all features. Table 2 compares feature selection by univariate analysis combined with fMRMR against fMRMR alone. Table 3 summarizes the results acquired with fMRMR feature selection and naive Bayes classification with leave-one-image-out and three-fold cross-validation. Table 4 summarizes the results acquired with fMRMR feature selection and SVM classification with leave-one-image-out and three-fold cross-validation. Among all predictors, ACM2 provides the best performance with fMRMR feature selection and naive Bayes classification (AUC = 0.90 and *Ac* = 82.86% for leave-one-image-out step). ROC curves, obtained for different feature sets with fMRMR feature selection and naive Bayes classification, are shown in Fig 4. For all models, obtained specificities were higher than the corresponding sensitivities, suggesting that we can identify patients with poor prognosis (the patients who should delay surgical resection in favor of aggressive systemic treatment).

**Table 2:** The area under ROC, classification accuracy (as a percentage), sensitivity, and specificity obtained with the proposed method using leave-one-image-out technique. The maximum *AUC* and *Ac* were highlighted with bold face. ‘***’ corresponds no outcome due to no features selected.

Feature Set	Univariate+fMRMR Feature Selection				fMRMR Feature Selection			
	AUC	*Ac*	*Sn*	*Sp*	AUC	*Ac*	*Sn*	*Sp*
GLCM	0.58	62.86	0.47	0.75	0.66	62.86	0.47	0.75
RLM	0.58	65.71	0.40	0.85	0.68	68.57	0.47	0.85
LBP	0.52	45.71	0.40	0.50	0.50	54.29	0.33	0.70
FD1	0.71	71.43	0.60	0.80	0.72	74.29	0.67	0.80
FD2	***	***	***	***	0.54	54.29	0.33	0.70
IH	0.65	65.71	0.47	0.80	0.69	68.57	0.47	0.85
ACM1	0.77	71.43	0.60	0.80	0.77	68.57	0.60	0.75
ACM2	0.88	80.0	0.67	0.90	**0.90**	**82.86**	**0.67**	**0.95**
All	0.84	68.57	0.53	0.80	0.83	74.29	0.60	0.85

**Table 3:** The area under ROC, classification accuracy (as a percentage), sensitivity, and specificity obtained with fMRMR feature selection and naive Bayes classification using leave-one-image-out and three-fold cross-validation techniques. The maximum AUC and *Ac* are highlighted with bold face.

Feature Set	Leave One Image Out				Three-fold Cross Validation			
	AUC	*Ac*	*Sn*	*Sp*	AUC	*Ac*	*Sn*	*Sp*
GLCM	0.66	62.86	0.47	0.75	0.58	58.14	0.44	0.69
RLM	0.68	68.57	0.47	0.85	0.60	64.14	0.44	0.80
LBP	0.50	54.29	0.33	0.70	0.53	52.43	0.42	0.60
FD1	0.72	74.29	0.67	0.80	0.69	70.0	0.58	0.79
FD2	0.54	54.29	0.33	0.70	0.57	56.0	0.59	0.54
IH	0.69	68.57	0.47	0.85	0.61	60.71	0.44	0.73
ACM1	0.77	68.57	0.60	0.75	0.69	65.86	0.58	0.72
**ACM2**	**0.90**	**82.86**	**0.67**	**0.95**	**0.80**	**75.0**	**0.68**	**0.80**
All	0.83	74.29	0.60	0.85	0.68	65.43	0.55	0.73

**Table 4:** The area under ROC, classification accuracy (as a percentage), sensitivity, and specificity obtained with fMRMR feature selection and SVM classification using leave-one-image-out and three-fold cross-validation techniques. The maximum AUC and *Ac* are highlighted with bold face.

Feature Set	Leave One Image Out				Three-fold Cross Validation			
	AUC	*Ac*	*Sn*	*Sp*	AUC	*Ac*	*Sn*	*Sp*
GLCM	0.54	62.86	0.27	0.90	0.57	57.57	0.36	0.74
RLM	0.66	60.00	0.20	0.85	0.62	61.71	0.33	0.84
LBP	0.55	57.14	0.40	0.70	0.51	52.0	0.38	0.62
FD1	0.64	65.71	0.47	0.80	0.65	63.14	0.42	0.79
FD2	0.58	57.14	0.40	0.70	0.61	62.14	0.39	0.80
IH	0.51	60.00	0.27	0.85	0.58	60.86	0.36	0.79
ACM1	0.75	68.57	0.60	0.75	0.79	70.57	0.55	0.83
**ACM2**	**0.78**	**65.71**	**0.47**	**0.80**	**0.81**	**72.29**	**0.59**	**0.82**
All	0.77	77.14	0.73	0.80	0.67	62.29	0.46	0.75

We investigated whether inclusion of clinical variables in our model improves classification accuracy. Incorporating tumor volume and CA 19-9 level (two variables known prior to treatment, used in prognostic models [11]) resulted in no improvement in survival prediction. We did not investigate the use of pathologic variables as these are unavailable prior to surgery.

Table 5 summarizes features selected with >0.5 probability, indicating features selected most often by the model. In general, similar features were selected by leave-one-image-out and three-fold cross-validation steps.

**Table 5:** List of features selected with >0.5 probability by the model.

Feature Set	Selected Features	
	Leave-one-image-out	Three-fold cross-validation
GLCM	*G*_6_, *G*_8_, *G*_16_, *G*_17_	*G*_6_, *G*_16_,*G*_17_
RLM	*R*_4_, *R*_8_, *R*_10_	*R*_2_, *R*_4_, *R*_10_
LBP	*L*_26_, *L*_28_, *L*_29_, *L*_33_, *L*_44_, *L*_52_,*L*_119_	*L*_21_, *L*_26_, *L*_33_, *L*_36_, *L*_52_,*L*_119_
FD1	*F*1_3_, *F*1_4_, *F*1_6_, *F*1_7_, *F*1_17_, *F*1_23_,*F*1_27_, *F*1_35_	*F*1_1_, *F*1_3_,*F*1_4_,*F*1_6_,*F*1_23_, *F*1_27_
FD2	*F*2_2_, *F*2_4_, *F*2_6_	*F*2_1_, *F*2_2_, *F*2_4_,*F*2_5_
IH	*I*_1_, *I*_2_, *I*_3_	*I*_1_, *I*_2_, *I*_3_
ACM1	*A*_6_, *A*_10_	*A*_6_, *A*_10_
ACM2	*M*_2_, *M*_4_, *M*_6_, *M*_10_, *M*_11_, *M*_15_	*M*_2_, *M*_4_, *M*_6_,*M*_7_,*M*_8_ *M*_10_, *M*_11_,*M*_15_
All	*R*_10_, *L*_44_, *L*_119_, *F*1_3_, *F*1_6_,*F*1_27_, *F*1_35_, *F*2_2_, *M*_2_, *M*_4_,*M*_6_, *M*_10_,*M*_11_, *M*_15_	*F*2_2_, *M*_2_, *M*_4_,*M*_6_, *M*_10_,*M*_11_, *M*_15_

## Discussion

We demonstrate 2-year prediction of survival of pancreas cancer patients using texture image features extracted from pre-treatment CT scans. Our results indicate that there is important prognostic information to be leveraged in the images of pancreatic tumors. This is a clinically important problem because we are currently unable to distinguish patients with occult metastatic disease who would benefit from aggressive chemotherapy from those who could be immediately resected.

ACM1 and ACM2 achieved the best performance among all features and were selected most often. These features represent directional change in intensity (i.e., directional edge patterns) of an image. Fig 3 demonstrates differences in tumor and texture appearance for patients surviving >2 years and <2 years. ACM1 and ACM2 differ in appearance in the two study groups. Radiographically, differences in ACM may reflect areas of necrosis within the tumor with decreased enhancement on CT. These would have developed before the administration of neoadjuvant therapy, due to underlying histologic or genetic alterations. Within all ACM2 features that were extracted from the orientation image, six features, contrast (*M*_2_), variance (*M*_4_), sum average (*M*_6_), difference variance (*M*_10_), difference entropy (*M*_11_), and inertia (*M*_15_) of orientation patterns, were selected most frequently, whereas energy, correlation coefficient, inverse difference, Shannon entropy, information-theoretic measures of correlation, maximal correlation coefficient, Renyi entropy, and Tsallis entropy were never selected. Intensity-based (GLCM, RLM, LBP, FD1, FD2, and IH) features were not as effective as the edge-based features (ACM1 and ACM2): FD1 achieved best AUC of 0.72 and *Ac* of 74.29% with leave-one-image-out technique and AUC of 0.69 and *Ac* of 70.0% with three-fold cross-validation, among all the intensity descriptors. The combination of all features deteriorated performance (Table 3), likely due to overfitting (fitting 255 features to 35 patients).

The higher specificity obtained by our methods indicates more reliable prediction for patients alive more than 2 years, suggesting that texture analysis of PDAC may represent underlying biological differences apparent clinically. The actual biological differences between resectable PDAC that explain the variable patient outcomes is not well elucidated; however, newly identified genetic drivers and tumor-stromal interactions may provide a rationale for the observed tumoral texture differences [50, 51]. A current limitation of our present study is the necessity of manually delineating tumors from CT images. A radiologist was necessary for the tumor segmentation. The radiologist was blinded to clinical outcome, eliminating the potential for introducing bias into the segmentation of tumor volumes. Patients received the same imaging protocol as part of the clinical trial: the impact of imaging protocol variation on texture features is an open problem under investigation by many groups. Moreover, the study suffers from the small dataset and lack of external data for validation. We plan to address these limitations in future work by relating our texture features with genomics, expanding our patient cohort, and studying with multiple readers to address the impact of tumor volume variability on extracted features and classification results.

Importantly, we demonstrate that texture information extracted from pre-treatment CT images obtained under controlled clinical trial conditions has the potential to predict survival of PDAC patients. The comparative study of texture features with clinical variables shows the superiority of texture information over previously available measures. This result has significant clinical implications because there are no known pre-treatment prediction tools for PDAC. Prediction prior to treatment would enable optimal selection of patients for surgery or neoadjuvant chemotherapy and provides further insight into this disease. CT is the standard imaging modality used in the clinical staging of PDAC [52] so our proposed techniques may provide non-invasive disease surveillance in any medical center.

## Conclusion

The present study demonstrates that texture features extracted from pre-treatment CT can predict 2-year survival, a critical treatment time point in the clinical course of patients with PDAC. Across all features, directional edge-based ACM2 provides best performance with an AUC of 0.90 and 0.80 and *Ac* of 82.86% and 75.0% with the leave-one-image out and three-fold cross-validation techniques, respectively. The observed efficacy of edge-based features establishes an association between directional-edge patterns and patient survival. Prior to the use of these features in a prospective clinical trial, validation in a larger independent cohort is required. Work is in progress to explore associations of texture with genetic sequencing, histology, and stromal content in an independent data set.

## Supporting information

S1 Table List of 255 imaging features used in analysis. (PDF)
