# Supplementary material for: Preliminary study of tumor heterogeneity in imaging predicts two year survival in pancreatic cancer patients
Source: PLoS One. 2017 Dec 7;12(12):e0188022. doi: 10.1371/journal.pone.0188022 (PMC5720792; doi:10.1371/journal.pone.0188022)
Supplement: S1 Table — (PDF) [file pone.0188022.s001.pdf]

S1 Table: List of 255 imaging features used in analysis.

**A.**	**GLCM (19 features) [26,33,34]**<br>1. Energy<br>2. Contrast<br>3. Correlation<br>4. Sum of squares<br>5. Inverse difference moment<br>6. Sum average<br>7. Sum variance<br>8. Entropy<br>9. Difference variance<br>10. Sum entropy<br>11. Difference entropy<br>12. Information-theoretic measures of correlation 1<br>13. Information-theoretic measures of correlation 2<br>14. Maximum correlation coefficient<br>15. Inertia<br>16. Cluster shade<br>17. Cluster prominence<br>18. Renyi entropy<br>19. Tsallis entropy
**B.**	**RLM (11 features) [27]**<br>1. Short run emphasis<br>2. Long run emphasis<br>3. Gray-level nonuniformity<br>4. Run length nonuniformity<br>5. Run percentage<br>6. Low gray-level run emphasis<br>7. High gray-level run emphasis<br>8. Short run low gray-level emphasis<br>9. Short run high gray-level emphasis<br>10. Run low gray-level emphasis<br>11. Long run high gray-level emphasis
**C.**	**LBP (128 features) [28,29,37]**<br>1. 59 unique output levels of uniform LBP (ULBP)<br>2. 10 unique output levels of rotation invariant (RI) ULBP<br>3. Standard deviation (SD), skewness, kurtosis, and entropy of ULBP histogram<br>4. SD, skewness, kurtosis, and entropy of RI-ULBP histogram<br>5. Mean, SD, skewness, kurtosis, and entropy of LBP histogram<br>6. SD, skewness, kurtosis, and entropy of efficient RI-LBP histogram<br>7. SD, skewness, kurtosis, and entropy of rotated LBP histogram<br>8. 38 Fourier descriptors of RI-ULBP histogram
**D.**	**FD1 (48 features) [41]**<br>1. FD from each of the 16 binary images<br>2. Mean gray value from each of the 16 binary images<br>3. Pixel count from each of the 16 binary images
**E.**	**FD2 (6 features) [39]**<br>1. Maximum of mean of FD<br>2. Maximum of standard deviation of FD<br>3. Maximum of lacunarity of FD<br>4. Average of mean of FD<br>5. Average of standard deviation of FD<br>6. Average of lacunarity of FD
**F.**	**IH (5 features)**<br>1. Mean<br>2. Standard deviation<br>3. Skewness<br>4. Kurtosis<br>5. Entropy

Continued on next page

Table S1–*Continued from previous page*

**G.**	**ACM1 and ACM2 (total 38 features, 19 from each)**	**[31, 32]**
	1. Energy	
	2. Contrast	
	3. Correlation	
	4. Sum of squares	
	5. Inverse difference moment	
	6. Sum average	
	7. Sum variance	
	8. Entropy	
	9. Difference variance	
	10. Sum entropy	
	11. Difference entropy	
	12. Information-theoretic measures of correlation1	
	13. Information-theoretic measures of correlation2	
	14. Maximal correlation coefficient	
	15. Inertia	
	16. Cluster shade	
	17. Cluster prominence	
	18. Renyi entropy	
	19. Tsallis entopy	
